# Effective dose from external radiation and ^222^Rn exhalation of construction materials manufactured with carbonated alkaline waste materials

**DOI:** 10.1007/s11356-026-37752-6

**Published:** 2026-04-20

**Authors:** José Antonio Suárez-Navarro, Ana María Moreno-Reyes, Víctor Manuel Expósito-Suárez, Ana Guerrero, Moisés Frías, Guillermo Hernáiz, José Francisco Benavente

**Affiliations:** 1 CIEMAT: Centro de Investigaciones Energeticas Medioambientales y Tecnologicas (Environment), Avd. Complutense 40, Madrid, 28040 Spain; 2 Eduardo Torroja Institute for Construction Sciences (IETcc-CSIC), Madrid, 28033 Spain

**Keywords:** Supplementary cementitious materials, Biomass ash, Alkaline waste, Gamma spectrometry, ^222^Rn emanation

## Abstract

The use of alkaline waste in construction enables dual CO_2_ removal, both through carbonation processes employed to enhance their properties as construction materials and by substituting them in cement production. However, despite their environmental benefits and demonstrated properties such as strength, water absorption, chemical reactivity, porosity, etc., these materials may contain natural radionuclides at elevated activity concentrations. Therefore, it is necessary to analyse these materials to ensure their safety from a radiological protection perspective for humans and the environment. The alkaline supplementary cementitious materials (SCMs) studied were a white steel slag (LFS), a biomass ash (BA), and a siliceous construction and demolition waste (CDW). This study presents the radiological characterisation via gamma spectrometry of the individual materials, hardened cement pastes fabricated with 80% ordinary Portland cement (OPC) and 20% of these alkaline SCMs, and ground hardened pastes. The alkaline residue BA exhibited high concentrations of ^210^Pb and ^40^K (around 5000 Bq kg^−1^), both with and without accelerated carbonation. However, the absorbed dose rates from external radiation of the final construction materials were lower than the natural background radiation level of 84 nGy h^−1^. On the other hand, the emanation and exhalation rates of ^222^Rn, both in the individual materials and in the cement pastes containing 20% of the SCMs, were equivalent to those obtained with OPC cement.

**Graphical abstract:**

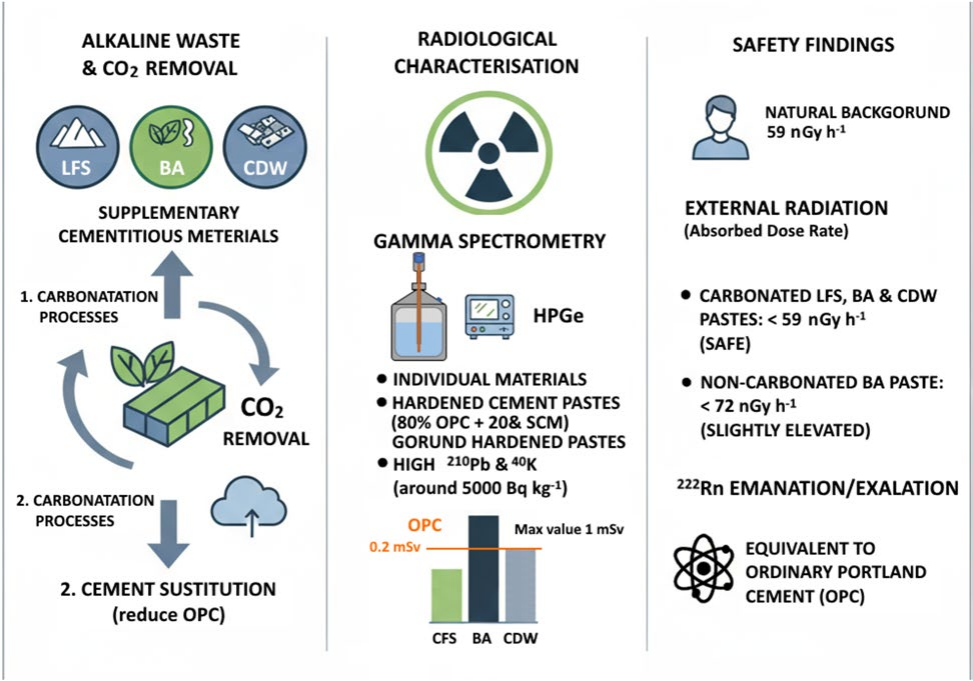

## Introduction

The investigation of procedures to reduce CO_2_ emissions produced during the manufacturing of construction materials (clinker, metakaolin, etc.) is a common objective for the scientific community. Estimates of CO_2_ emissions range between 800 and 1000 kg per tonne of cement produced (Abiodun et al. 2022). This amount of CO_2_ accounts for 5–8% of total emissions (Ige et al. 2024). Reductions involve the use of new clinker production technologies such as: (i) waste heat recovery (WHR) (Kunche and Mielczarek 2021) , (ii) alternative clinker technologies like Calcium Sulfoaluminate/Belite-Ye’elimite-Ferrite (CSA/BYF), Solidia, Calitement, and X-Clinker, which allow emissions reductions of 30% to 50% (Antunes et al. 2022); (iii) alkali-activated cements with fly ash, metakaolin, etc. (Barboza-Chavez et al. 2020), and (iv) the addition of supplementary cementitious materials (SCMs) such as silica fume, fly ash, or slags to cement, which could reduce CO_2_ emissions by 15% to 30% (Yang et al. 2015). Currently, one of the emerging and innovative approaches to complying with the cement industry’s Roadmap (5Cs) to achieve climate neutrality by 2025 (Abiodun et al. 2022; Ige et al. 2024) is to use alkaline industrial waste as CO_2_ sinks before using it as SCMs to manufacture eco-cement with a lower carbon footprint and greater sustainability. Currently, work is focused on unconventional and non-standardised alkaline waste (fly ash, blast furnace slag, metakaolin). In this regard, previous work by some of the co-authors of this study has focused on generating new scientific and technical knowledge relevant to both the scientific community and the construction sector. Previous studies involve the accelerated carbonation of alkaline wastes such as white ladle furnace slag (LFS), biomass ash (BA), and fine concrete waste fraction (CDW), which can replace between 7 and 20% of cement (Frías et al. 2024a), with the added advantage that their accelerated carbonation allows for the absorption of a percentage of CO_2_. LFS, BA, and CDW contain different phases such as portlandite (Ca(OH)_2_), periclase (MgO), olivine (Ca_2_SiO_4_), along with other silicates and aluminates. CO_2_ reacts with Ca(OH)_2_ to form calcium carbonate (calcite (CaCO_3_)), reducing alkalinity and stabilising the material. MgO reacts with CO_2_ and H_2_O, producing hydrated magnesium carbonates (brucite or dolomite), which stabilise the phase and prevent expansions. Olivine partially transforms during carbonation, releasing amorphous silica that forms CSH, C(A)SH gels and carbonates (Frías et al. 2024b). These reactions result in more compact final structures free of cracks, leading to increased mechanical strength (Moreno de los Reyes et al. 2025).

Therefore, the use of alkaline wastes implies both improvements in the characteristics of construction materials (strength, water absorption, chemical reactivity, porosity arrangement, etc.) and a dual reduction in CO_2_, as they not only reduce the amount of cement but also serve to absorb CO_2_ through carbonation reactions. However, the use of alkaline materials such as steel slags and fly ash raises the need to investigate the presence of natural radionuclides, as their generation processes can lead to the concentration of the raw materials used, potentially resulting in an enrichment of natural radionuclides. Granulated blast furnace slags often contain ^226^Ra levels exceeding 100 Bq kg^−1^, which may result in effective dose rates from external radiation that must be verified (Khobotova et al. 2021). Additionally, biomass ashes also concentrate ^40^K and ^210^Pb at activity concentrations significantly higher than 3000 Bq kg^−1^, which could lead to an excess annual effective dose rate above 1 mSv (Ondrasek et al. 2023). Therefore, it is essential to ensure the proper use of these materials from the perspective of radiological protection despite their properties as construction materials. Furthermore, previous studies have observed that the carbonation of Portland cement mortars with granulated blast furnace slag (GGBFS) caused a ^214^Pb/^214^Bi disequilibrium with emanation factors (*F*) exceeding a fraction of 0.25 (Sanjuan et al. 2022). Thus, it is necessary to understand the potential increase in the emanation of powdered materials, both anhydrous and hydrated, and the radon exhalation rate of the final hardened materials.

Based on the above, the purpose of this study was to verify that carbonated alkaline materials, even though they may contain high activity concentrations of natural radionuclides, do not exceed an annual effective dose rate from external radiation higher than 1 mSv, and that both the emanation and exhalation of ^222^Rn are comparable to those of ordinary Portland cement (OPC). The tasks necessary to verify our main objective were as follows: (i) radiologically characterise the anhydrous powdered samples, the hardened hydrated samples in 5 × 5 × 5 cm^3^ cubes, and these milled samples using gamma spectrometry, (ii) correlate the chemical compositions with the activity concentrations of natural radionuclides, and (iii) determine the annual effective dose rate, as well as the ^222^Rn emanation of the powdered materials and the exhalation rate of the hardened cubes.

## Materials, methods, and equipment

### Construction materials

#### Individual materials

The OPC cement used in this study was of type CEM I 52.5R, supplied by Cementos Lemona, S.A. (Bilbao, Spain), and complies with the requirements of the European Standard EN 197:1 (AENOR 2011). The alkaline wastes used were: (i) white steel slag sourced from northern Spain (Basque Country) with a particle size below 4 mm (LFS), (ii) a fine fraction of siliceous concrete waste (CDW) less than 5 mm, and (iii) biomass ash generated from forest pruning and cereal residues (BA) sourced from Extremadura (Spain). These materials were investigated in previous studies, allowing confirmation of their suitability and behaviour in the carbonation processes applied in this work.

#### Carbonation method for alkaline wastes

The carbonation of the three types of alkaline wastes was carried out at the facilities of Tecnalia (Bilbao) in a static 5 L reactor with a capacity of 5 kg, characteristic of a laboratory-scale reactor (Frías et al. 2024b). The carbonation conditions were as follows: (a) humidity levels of 15% for LFS, 30% for BA, and 10% for CDW, (b) 100% dry CO_2_, (c) particles with a size smaller than 4 mm, and (d) pressure and temperature conditions of 1 bar and 20 °C. The weight increase of the formed carbonates was determined after 90 min using the calcimetry test UNE 103200 (AENOR 2021). The CO_2_ equivalents fixed per kilogram for each waste under these conditions were BA 60.5 eq. CO_2_ kg^−1^, LFS 42.7 eq. CO_2_ kg^−1^, and CDW 6.8 eq. CO_2_ kg^−1^. Subsequently, the carbonated wastes were dried at 105 °C for 24 h. The final carbonated wastes were designated as LFSc, BAc, and CDWc. All carbonate and non-carbonated waste was ground and sieved to below 45 μm before being added as SCMs.

#### Blended cement pastes

The blended cement pastes were produced in cubic specimens of 5 × 5 × 5 cm^3^ using a 20% partial replacement of the three alkaline wastes, both carbonated and non-carbonated, with a water-to-cement ratio of 0.5, in accordance with ASTM C109 (International 2016). Subsequently, the cubes were submerged in water for the 28 days required for curing the specimens.

The hardened cement pastes were also ground using an agate ball mill (Retsch model RM 200).

### Gamma spectrometry

The samples used in this study were measured using two HPGe gamma detectors of the XtRa type, with relative efficiencies of 42.1% and 115.7%. Both detectors had a resolution of 2 keV for the 1173 keV photopeak of ^60^Co. The detectors were equipped with passive Pb shielding with a thickness of 15 cm, along with two layers of Cu and Zn of 1 mm each to prevent X-rays from Pb. The detectors were connected to a DSA-LX module, which integrated the detector’s associated electronics: high-voltage power supply, amplifier, ADC, and communication module with the computer (MIRION 2017). Spectra were acquired and analysed using Genie 2000 software (CANBERRA 2002). The true coincidence summing effect was corrected using the Peak-To-Total algorithm, as radionuclides such as ^214^Bi, ^228^Ac, ^208^Tl, and ^235^U are subject to this type of coincidence (CANBERRA 2008). ^226^Ra was directly determined from the 186 keV photopeak using the method described in (Papachristodoulou et al. 2003). The energies and emission probabilities of the radionuclides analysed in this study were the following (Be et al. 2016): ^234^Th (63.30 (2) keV–3.75 (8)%), ^226^Ra (186.211 (13) keV–3.555 (19)%), ^214^Pb (351.932 (2) keV–35.60 (7)%), ^214^Bi (609.312 (7) keV–45.49 (19)%; 1120.287 (10) keV–14.91 (3)%; 1764.494 (14) keV–15.31 (5)%), ^210^Pb (46.539 (1) keV–4.252 (40)%), ^212^Pb (238.632 (2) keV–43.6 (5)%), ^208^Tl (583.187 (2) keV–85.0 (3)%), ^228^Ac (911.196 (6) keV–26.2 (8)%), ^235^U (163.356 (3) keV–5.08 (3)%; 205.16 (4) keV–5.02 (3)%; 143.767 (3) keV–10.94 (6)%), ^40^K (1460.822 (6) keV–10.55 (11)%), and ^137^Cs (661.657 (3) keV–84.99 (20)%).

The detectors were calibrated in energy (*E*) as a function of channel (Ch) using a gamma cocktail containing the following radionuclides (Be et al. 2016): ^210^Pb (46.539 (1) keV), ^241^Am (59.5409 (1) keV), ^109^Cd (88.0336 (10) keV), ^57^Co (122.06065 (12) keV), ^139^Ce (165.8575 (11) keV), ^113^Sn (391.698 (3) keV), ^60^Co (1173.228 (3) keV and 1332.492 (4) keV), and ^88^Y (898.042 (11) keV and 1836.070 (8) keV). The fitting function used was a linear equation: $E=a\times Ch+b$, where *a* and *b* are the fitting parameters. Additionally, the efficiency calibration (*ε*) as a function of energy was calculated using the LabSOCS code, as the two detectors used were characterised by Mirion-Canberra. The analytical function employed for fitting the efficiency curve was: $\epsilon =\sum_{i}{P}_{i}(\mathrm{ln}{E}_{\gamma }{)}^{i-1}$, where *P*_*i*_ are the fitting parameters of the curve (Oladipo 2001).

The geometries used to measure the samples were of two types: (i) powdered samples (individual and milled cubes) placed in cylindrical polypropylene containers with a diameter of 76 mm and a height of 30 mm and (ii) hardened paste cubes of 5 × 5 × 5 cm^3^. The samples were left to rest for 21 days to allow secular equilibrium to be reached between ^226^Ra and its short-lived progeny, ^214^Pb and ^214^Bi, through which the indirect calculation of the activity concentration of ^226^Ra was performed. The samples were measured for a minimum of 80,000 s, and background measurements were conducted for 600,000 s. The laboratory where the measurements were performed was accredited by ENAC (National Accreditation Body) in accordance with ISO/IEC 17025:2017 (UNE 2017).

### Effective dose rate

The European Directive 2013/59 on health protection against ionising radiation (EU 2013), transposed into Spanish legislation through RD 1029/2022 (BOE 2022), establishes the Activity Concentration Index (ACI) to assess the suitability of a construction material. Although this index is widely accepted within the scientific community, determining the annual effective dose using the model proposed by Nuccetelli et al. (2012) provides a more accurate assessment. This model defines the absorbed dose rates in air for ^226^Ra, ^232^Th, and ^40^K (nGy h^−1^ per Bq kg^−1^) based on a standard room model with dimensions of 5 × 4 × 2.8 m^3^, a wall thickness of 20 cm, and a density of 2.35 g cm^−3^. The equation to determine the absorbed dose rate from external radiation ($\dot{D}$, nGy h^−1^) is given by Expression 1:

1 $$\dot{D}=(0.08\times {C}_{{}^{40}K}+0.92\times {C}_{{}^{226}Ra}+1.1\times {C}_{{}^{232}Th})$$

where ${C}_{{}^{40}K}$, ${C}_{{}^{226}Ra}$, and ${C}_{{}^{232}Th}$ are the activity concentrations of ^40^K, ^226^Ra, and ^232^Th, respectively, in Bq kg^−1^. The coefficients 0.08, 0.92, and 1.1 are the effective dose rates (nGy h^−1^ per Bq kg^−1^) (Nuccetelli et al. 2012). The absorbed dose rate in air corresponding to the global background is 84 nGy h^−1^ (UNSCEAR 2008). The uncertainty associated with the absorbed dose rate is given by Expression 2:

2 $$u(\dot{D})=\sqrt{(0.08{)}^{2}\times {u}^{2}({C}_{{}^{40}K})+(0.92{)}^{2}\times {u}^{2}({C}_{{}^{226}Ra})+(1.1{)}^{2}\times {u}^{2}({C}_{{}^{232}Th})}$$

On the other hand, the annual effective dose rate (*E*_g_, mSv) is calculated using Expression 3:

3 $$\dot{{E}_{g}}=\dot{D}\times V\times {T}_{e}\times O\times {10}^{-6}$$

where *V* is the conversion factor from absorbed dose to effective dose (0.7 Sv Gy^−1^), *T*_e_ is the number of hours in a year (8760 h), *O* is the occupancy factor (0.8, corresponding to 80% occupancy), and 10^–6^ is the conversion factor from nano-to-milli. The uncertainty associated with $\dot{E}$ is given by Expression 4:

4 $$u(\dot{{E}_{g}})=V\times {T}_{e}\times O\times {10}^{-6}\times u(\dot{D})$$

The effective dose rate corresponding to the natural radioactive background is 0.48 mSv.

### Determination of ^222^Rn emanation and exhalation

The emanation and exhalation of ^222^Rn were determined using the AlphaGUARD DF2000 equipment from Bertin Technologies. Powdered samples were used to determine the emanation of ^222^Rn, while hardened paste samples of 5 × 5 × 5 cm^3^ were used to measure the exhalation of ^222^Rn. Both emanation and exhalation were measured using the accumulation method over a period of no fewer than 72 h. The samples were measured for 2 h after the accumulation period, following the procedure provided by the AlphaGUARD equipment to eliminate interference caused by ^220^Rn during the measurement. The emanation factor (*F*) was determined using Expression 5 (de With et al. 2017):

5 $$F=\frac{{\lambda }_{\mathrm{eff}}\times V\times {C}_{\mathrm{Max}}}{m\times {\lambda }_{{}^{222}Rn}\times {C}_{{}^{226}Ra}}$$

where ${\lambda }_{\mathrm{eff}}$ is the effective decay constant of ^222^Rn in s^−1^, *V* is the volume of the accumulation chamber in m^3^, *C*_Max_ is the equilibrium or saturation concentration of ^222^Rn in Bq m^−3^, *m* is the mass of the sample in kg, ${\lambda }_{{}^{222}Rn}$ is the decay constant of ^222^Rn in s^−1^, and ${C}_{{}^{226}Ra}$ is the activity concentration of ^226^Ra in the sample in Bq kg^−1^. The ${\lambda }_{\mathrm{eff}}$ is defined as ${\lambda }_{v}+{\lambda }_{{}^{222}Rn}$, where ${\lambda }_{v}$ is the ventilation rate in s^−1^. The uncertainty associated with the emanation factor (*u*(*F*)) is given by Expression 6:

6 $${u}^{2}(F)={\left[{\omega }_{C(t)}\right]}^{2}\times {u}^{2}\left[C(t)\right]+{\left[{\omega }_{C(0)}\right]}^{2}\times {u}^{2}\left[C(0)\right]+{\left[{\omega }_{{C}_{{}^{226}Ra}}\right]}^{2}\times {u}^{2}\left[{C}_{{}^{226}Ra}\right]$$

where *C*(*t*) is the activity concentration of ^222^Rn at time t of accumulation in Bq m^−3^, and *C*(0) is the activity concentration of ^222^Rn at time *t* = 0. Furthermore:

7 $${\omega }_{C(t)}=\frac{{\lambda }_{\mathrm{eff}}\times V}{m\times {\lambda }_{{}^{222}Rn}\times {C}_{{}^{226}Ra}}\times \frac{1}{1-{e}^{-{\lambda }_{\mathrm{eff}}\times t}}$$

8 $${\omega }_{C(0)}=\frac{{\lambda }_{\mathrm{eff}}\times V}{m\times {\lambda }_{{}^{222}Rn}\times {C}_{{}^{226}Ra}}\times \frac{-{e}^{-{\lambda }_{\mathrm{eff}}\times t}}{1-{e}^{-{\lambda }_{\mathrm{eff}}\times t}}$$

9 $${\omega }_{{C}_{{}^{226}Ra}}=\frac{-{\lambda }_{\mathrm{eff}}\times V}{m\times {\lambda }_{{}^{222}Rn}\times {C}_{{}^{226}Ra}^{2}}\times \frac{C(t)-C(0)\times {e}^{-{\lambda }_{\mathrm{eff}}\times t}}{1-{e}^{-{\lambda }_{\mathrm{eff}}\times t}}$$

The detection limit of the emanation factor was determined using the criteria outlined in ISO 11929 (ISO 2005). First, the decision threshold (F*) was calculated, considering the condition that $C(t)\approx C(0)\times {e}^{-{\lambda }_{\mathrm{eff}}\times t}$, and where *u*(0) is the uncertainty of the emanation factor when its value is 0. The resulting expression to determine the decision threshold was:

10 $${F}^{*}={K}_{1-\beta }\times u(0)$$

where ${K}_{1-\beta }$ is the critical value of the standard normal distribution, equal to 1.645. From the decision threshold, the detection limit (*F*^#^) was determined, as given by Expression 11:

11 $${F}^{\#}=\frac{A+\sqrt{{A}^{2}-4{k}_{1-\beta }^{2}{\sigma }_{Ra}^{2}\times \left[({F}^{*}{)}^{2}-{k}_{1-\beta }^{2}S(t)\right]}}{2{k}_{1-\beta }^{2}{\sigma }_{Ra}^{2}}$$

where *A*, $\Phi (t)$, *S(t)*, and ${\sigma }_{\mathrm{Ra}}^{2}$ are given by the following expressions:

12 $$A=2{F}^{*}+{k}_{1-\beta }^{2}{\omega }_{{C}_{\mathrm{max}}^{\#}}^{2}\Phi (t)$$

13 $$\Phi (t)=\frac{m{\lambda }_{{}^{222}{\mathrm{Rn}}}{C}_{{}^{226}{\mathrm{Ra}}}}{{\lambda }_{\mathrm{eff}}V}\left(1-{e}^{-{\lambda }_{\mathrm{eff}}t}\right)$$

14 $$S(t)=C(0){e}^{-{\lambda }_{\mathrm{eff}}t}{\omega }_{{C}_{\mathrm{max}}^{\#}}^{2}+{\omega }_{C(0)}^{2}{u}^{2}[C(0)]$$

15 $${\sigma }_{Ra}^{2}=\frac{{u}^{2}[{C}_{{}^{226}Ra}]}{{C}_{{}^{226}Ra}^{2}}$$

The exhalation factor (*E*, mBq s^−1^ m^−2^) was determined using Expression 16 (Hassan 2014):

16 $$E=\frac{{\lambda }_{\mathrm{eff}}\times V}{S}\times \frac{C(t)-C(0)\times {e}^{-{\lambda }_{\mathrm{eff}}\times t}}{1-{e}^{-{\lambda }_{\mathrm{eff}}\times t}}$$

where *S* is the surface area of the sample in m^2^. The uncertainty of *E* is defined as:

17 $${u}^{2}(E)={\left[{\omega }_{C(t)}\right]}^{2}\times {u}^{2}\left[C(t)\right]+{\left[{\omega }_{C(0)}\right]}^{2}\times {u}^{2}\left[C(0)\right]$$

Furthermore, the decision threshold for *E* is given by Expression 18, and the detection limit by Expression 19:

18 $${E}^{*}={K}_{1-\beta }\times u(0)$$

19 $${E}^{\#}=\frac{A+\sqrt{{A}^{2}-4({E}^{*}{)}^{2}+4{k}_{1-\beta }^{2}S(t)}}{2}$$

where *A* and $\Psi (t)$ are given by the following expressions:

20 $$A=2{E}^{*}+{k}_{1-\beta }^{2}{\omega }_{{C}_{\mathrm{max}}^{\#}}^{2}\Psi (t)$$

21 $$\Psi (t)=\frac{S}{{\lambda }_{\mathrm{eff}}V}\left(1-{e}^{-{\lambda }_{\mathrm{eff}}t}\right)$$

## Results and discussion

### Radioactive content of the different individual materials

Table 1 presents the activity concentrations of radionuclides belonging to the natural radioactive series of uranium, actinium, and thorium, along with ^40^K and ^137^Cs as anthropogenic radionuclides. The activity concentrations reveal some noteworthy aspects for each of the tested materials. The LFS sample shows an absence of ^40^K, as well as a disequilibrium in the case of ^210^Pb, with a ratio of 2.57 for ^234^Th/^210^Pb. These results are consistent with those obtained in previous studies on various steel slags (Ibrahiem et al. 2000). Potassium is present at 0.07% in steel slags due to its mobility, which prevents its retention in the slag (Al-Kawari and Hushari 2019, Zhao et al. 2022). Furthermore, the ^234^Th/^210^Pb disequilibrium is attributed to the volatilisation of lead at the furnace temperature, which is transported by fine particles, thereby reducing its presence in the furnace slag (Mohiuddin et al. 2014). The BA sample exhibits high concentrations of ^210^Pb, ^40^K, and ^137^Cs, which is the expected behaviour for this type of ash (Radulovic and Pepin 2023). These results are due to the concentration caused by the combustion of wood from different plant species, which absorb natural and anthropogenic atmospheric radionuclides. The ^210^Pb originates from atmospheric ^222^Rn and is transported in submicrometric particles that are absorbed through the stomata of leaves (Uddin et al. 2024). Submicrometric particles also transport ^137^Cs, derived from soil resuspension processes. Resuspended ^137^Cs is deposited on the stomata of plant leaves, leading to its absorption when it comes into contact with rainwater or dew (Kaste et al. 2021). Finally, the CDW sample exhibits the typical radioactive content observed in cement samples, which contribute the most radiological input to mortars and concrete (Lewicka et al. 2022).

**Table 1:** Activity concentrations (Bq kg^−1^) of natural radionuclides belonging to the natural radioactive series of uranium, actinium, and thorium, along with ^40^K and the anthropogenic radionuclide ^137^Cs

Reference	^234^Th (Bq kg^−1^)	^226^Ra (Bq kg^−1^)	^214^Pb (Bq kg^−1^)	^214^Bi (Bq kg^−1^)	^210^Pb (Bq kg^−1^)	^235^U (Bq kg^−1^)	^228^Ac (Bq kg^−1^)	^212^Pb (Bq kg^−1^)	^208^Tl (Bq kg^−1^)	^40^K (Bq kg^−1^)	^137^Cs (Bq kg^−1^)
LFS	29.6 ± 6.7	27.4 ± 8.3	29.0 ± 2.6	27.5 ± 1.9	11.5 ± 7.8	< 3.0	8.6 ± 1.0	8.91 ± 0.87	3.41 ± 0.45	< 7.6	< 0.6
LFSc	24.2 ± 6.4	26 ± 10	13.4 ± 1.3	12.2 ± 1.3	< 9.1	< 2.9	4.76 ± 0.62	5.70 ± 0.84	2.9 ± 1.1	< 6.8	< 0.5
BA	< 23.4	< 19.0	8.4 ± 1.2	7.9 ± 1.1	5283 ± 448	< 5.4	5.4 ± 1.8	5.78 ± 0.71	2.21 ± 0.50	4155 ± 355	31.2 ± 2.8
BAc	< 20.9	12.5 ± 3.5	8.97 ± 0.93	8.2 ± 1.0	4838 ± 410	< 5.0	5.0 ± 1.0	5.9 ± 1.7	2.3 ± 1.2	4210 ± 361	31.3 ± 2.9
CDW	35 ± 10	20 ± 10	25.7 ± 2.4	23.0 ± 1.7	31 ± 10	< 3.4	22.6 ± 1.9	24.1 ± 2.1	8.55 ± 0.86	485 ± 43	< 0.8
CDWc	34 ± 10	16 ± 14	22.7 ± 2.0	21.9 ± 2.1	31.3 ± 9.4	< 3.2	21.4 ± 2.6	23.4 ± 2.4	8.0 ± 1.1	463 ± 42	< 0.5

The uncertainties are quoted for a coverage factor of *k* = 2

**Fig. 1:**
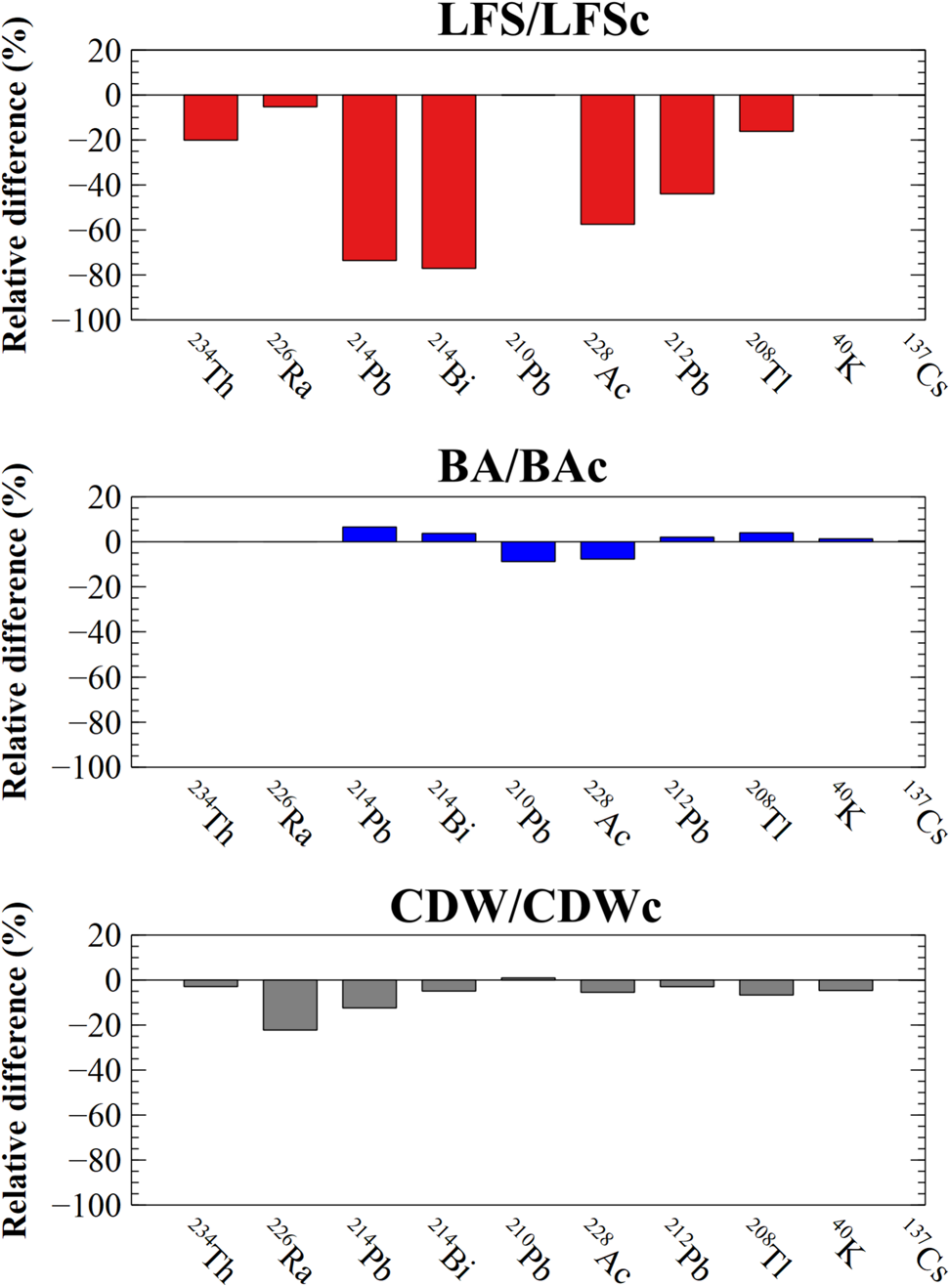
Relative differences in the activity concentrations of the various natural radionuclides and ^137^Cs in individual materials (LFS/LFSc, BA/BAc, and CDW/CDWc) with and without carbonation

The potential change in the activity concentrations of the radionuclides from the uranium, actinium, and thorium series, along with ^40^K, in the three types of samples after carbonation was determined by applying the paired-sample Student’s *t*-test for a two-tailed distribution with a significance level of *α* = 0.05. The *p*-value obtained when comparing the LFS and LFSc samples was less than 0.05 (0.04), indicating that the influence of carbonation is statistically significant. However, biomass ash and demolition waste did not exhibit this behaviour, with *p*-values of 0.41 and 0.15, respectively. Figure 1 shows the relative difference in the activity concentrations of natural radionuclides and ^137^Cs before and after carbonation of the samples. The LFS sample exhibited the greatest differences. The thorium series (^228^Ac, ^212^Pb, and ^208^Tl) was the most affected overall. However, the loss of reservoir radionuclides (^232^Th, ^228^Th, and ^228^Ra) could not be justified since the temperatures at which carbonation was performed would not account for the solubilisation of Th or Ra (Nisbet et al. 2022). Nevertheless, the potential pH change from 12 to 9 caused by carbonation could facilitate the release of Th, which, together with the formation of complexes with carbonate, would lead to the liberation of Th (Gijbels et al. 2019). Similarly, the ^226^Ra/^214^Pb disequilibrium would be equivalent to that observed in other studies (Kovler et al. 2017), which could be explained by a high emanation of ^222^Rn from the material. In this regard, Wang et al. (2020) demonstrated that the carbonation of steel slags induces significant microstructural changes, progressively eroding the original smooth surface of the particles until completely destroying their structure at high degrees of carbonation (52%) (Wang et al. 2020). These microstructural changes could account for both the loss of Th (reflected in the decrease of gamma descendants with short half-lives) and the ^226^Ra/^214^Pb disequilibrium observed in the case of LFSc. However, this behaviour was not observed in the samples where carbonation was not performed, as the activity concentrations of ^226^Ra, ^214^Pb, and ^214^Bi overlapped for a coverage factor *k* = 2. Therefore, there is an effect of radionuclide loss during the carbonation of LFS, which could be investigated further in future studies. The observed ^226^Ra/^214^Pb ratio could be attributed to a possible rearrangement of Ra atoms within the structure or to chemical changes. This could be justified by the observed decrease in CaO content after carbonation, which is more pronounced in LFSc and BAc, a behaviour reproduced in these materials compared to CDWc, where no decrease in CaO is observed (Moreno de los Reyes et al. 2025).

### Radioactive content and annual effective dose rate of hardened cement pastes

Table 2 presents the activity concentrations of radionuclides belonging to the uranium series (^234^Th, ^226^Ra, ^214^Pb, ^214^Bi, and ^210^Pb) and the thorium series (^228^Ac, ^212^Pb, and ^208^Tl), along with ^40^K and ^137^Cs. The table also displays the annual effective dose rate from external radiation (*E*_*g*_) determined based on the activity concentrations of ^226^Ra, ^212^Pb, and ^40^K. The activity concentrations obtained for hardened paste cubes made with 80% OPC and 20% of the alkaline supplementary cementitious material (LFS, BA, and CDW, both carbonated and non-carbonated) were consistent with the results obtained for the individual materials (Table 1). The activity concentrations of ^40^K and ^210^Pb in the hardened paste cubes made with 20% BA (both carbonated and non-carbonated) were higher than those in the other cubes. However, to verify whether significant differences existed amongst the results of the various samples, the means of each material were compared using the two-tailed paired-sample Student’s t-test, with a significance level of *α* = 0.05.

**Table 2:** Activity concentrations (Bq kg^−1^) of radionuclides belonging to the uranium series (^234^Th, ^226^Ra, ^214^Pb, ^214^Bi, and ^210^Pb) and the thorium series (^228^Ac, ^212^Pb, and ^208^Tl), along with ^40^K and ^137^Cs, and annual effective dose rate (mSv y^−1^) of hardened cement pastes

Type	Ref	^234^Th (Bq kg^−1^)	^226^Ra (Bq kg^−1^)	^214^Pb (Bq kg^−1^)	^214^Bi (Bq kg^−1^)	^210^Pb (Bq kg^−1^)	^228^Ac (Bq kg^−1^)	^212^Pb (Bq kg^−1^)	^208^Tl (Bq kg^−1^)	^40^K (Bq kg^−1^)	^137^Cs (Bq kg^−1^)	${\dot{E}}_{g}$ (mSv y^−1^)
Hardened cement pasta cubes	OPC	32 ± 10	27 ± 18	27.2 ± 3.4	26.6 ± 2.9	23.3 ± 4.9	8.4 ± 1.4	11.8 ± 1.8	4.5 ± 1.0	92 ± 16	< 0.48	0.222 ± 0.082
	LFS	21.9 ± 2.5	24.9 ± 4.3	24.0 ± 2.1	22.6 ± 1.4	15.5 ± 1.9	9.48 ± 0.79	9.19 ± 0.80	3.52 ± 0.35	48.1 ± 5.0	< 0.43	0.181 ± 0.020
	LFSc	23.0 ± 5.6	24.5 ± 9.3	23.0 ± 2.2	22.3 ± 1.5	16.7 ± 4.5	8.3 ± 1.3	9.3 ± 1.1	3.51 ± 0.53	41.0 ± 5.9	< 0.48	0.177 ± 0.042
	BA	23.3 ± 4.5	25.9 ± 5.8	23.7 ± 2.1	22.5 ± 1.5	736 ± 63	9.6 ± 1.0	10.30 ± 0.93	3.84 ± 0.43	464 ± 41	3.27 ± 0.38	0.355 ± 0.031
	BAc	26 ± 10	21 ± 13	22.5 ± 2.4	20.8 ± 2.0	617 ± 56	5.93 ± 0.90	9.7 ± 1.2	3.80 ± 0.76	354 ± 32	2.97 ± 0.61	0.286 ± 0.060
	CDW	26.8 ± 3.5	28.7 ± 5.7	27.6 ± 2.4	26.6 ± 1.7	23.5 ± 3.1	13.5 ± 1.2	14.6 ± 1.3	5.23 ± 0.53	139 ± 13	< 0.44	0.263 ± 0.027
	CDWc	27.0 ± 8.2	24 ± 11	24.8 ± 2.5	24.4 ± 2.1	23.1 ± 6.9	12.1 ± 1.2	12.8 ± 1.5	4.99 ± 0.76	116 ± 13	< 0.51	0.223 ± 0.051
Powder from the crushed cubes	OPC	31.1 ± 5.5	26.2 ± 7.0	25.6 ± 2.3	22.2 ± 1.6	19.9 ± 2.7	11.0 ± 1.2	10.6 ± 1.0	3.82 ± 0.46	78.3 ± 8.9	< 0.67	0.206 ± 0.032
	LFS	30.5 ± 7.6	28 ± 12	24.2 ± 2.3	23.0 ± 1.7	18.7 ± 8.2	9.8 ± 1.1	9.8 ± 1.0	3.43 ± 0.45	55.3 ± 7.3	< 0.57	0.201 ± 0.055
	LFSc	31 ± 15	19 ± 15	22.0 ± 3.3	20.9 ± 3.0	18.7 ± 8.1	9.5 ± 1.7	9.7 ± 1.8	3.55 ± 0.48	58 ± 17	< 0.53	0.161 ± 0.069
	BA	27.2 ± 6.4	27.2 ± 7.4	25.1 ± 2.3	23.7 ± 1.8	788 ± 67	10.3 ± 1.2	11.1 ± 1.0	3.81 ± 0.47	470 ± 42	3.09 ± 0.42	0.367 ± 0.038
	BAc	30.5 ± 5.5	25.9 ± 9.4	24.5 ± 2.5	23.9 ± 3.6	739 ± 69	8.3 ± 2.0	11.8 ± 2.4	4.1 ± 1.4	419 ± 46	3.2 ± 1.3	0.345 ± 0.048
	CDW	29.2 ± 3.6	28.0 ± 6.1	24.8 ± 2.2	23.6 ± 1.7	30.0 ± 4.6	14.5 ± 1.4	14.7 ± 1.3	5.21 ± 0.57	130 ± 13	< 0.61	0.257 ± 0.029
	CDWc	27.6 ± 4.5	31.0 ± 7.4	25.7 ± 2.4	24.9 ± 1.9	27.5 ± 7.0	13.8 ± 1.5	13.6 ± 1.3	5.29 ± 0.62	145 ± 15	< 0.72	0.270 ± 0.035

The uncertainties are quoted for a coverage factor of *k* = 2

**Fig. 2:**
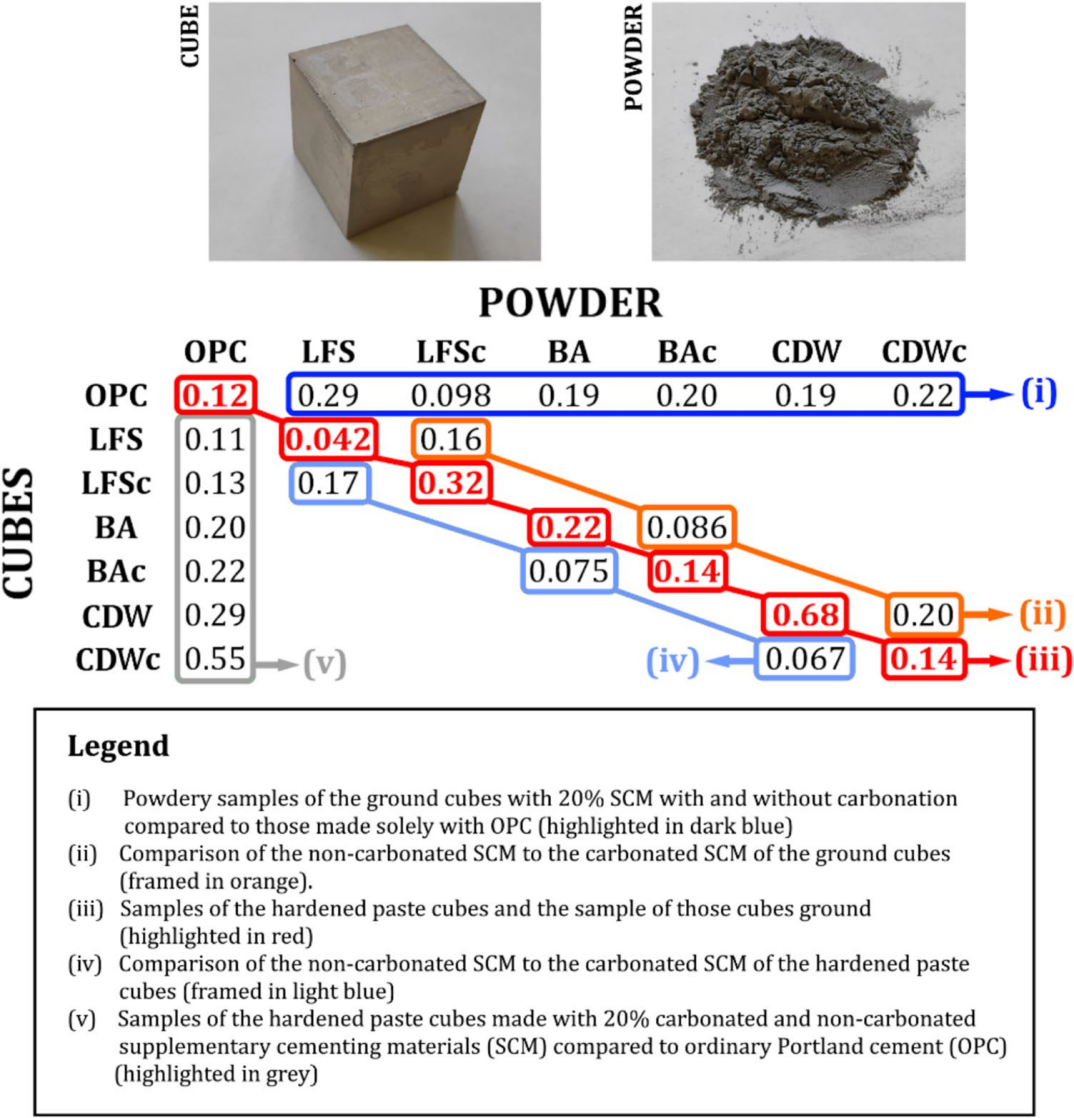
The results obtained by applying paired-sample Student’s *t*-tests to compare the activity concentrations of radionuclides belonging to the natural radioactive series of uranium, thorium, and ^40^K across different sets of results

Figure 2 shows the results obtained from applying paired-sample Student’s *t*-tests to compare the activity concentrations of radionuclides belonging to the natural radioactive series of uranium, thorium, and ^40^K from the following sets of results: (i) powdered samples from ground cubes containing 20% SCMs, both carbonated and non-carbonated, compared to those made solely with OPC (highlighted in dark blue); (ii) comparison of non-carbonated SCMs versus carbonated SCMs from ground cubes (framed in orange); (iii) samples from hardened paste cubes compared to ground samples of these cubes (highlighted in red); (iv) comparison of non-carbonated SCMs versus carbonated SCMs from hardened paste cubes (framed in light blue); and (v) samples from hardened paste cubes made with 20% of the supplementary cementitious materials (SCMs), both carbonated and non-carbonated, compared to those made with OPC (highlighted in grey). Comparisons were conducted as two-tailed tests to assess whether the means were statistically different. None of the comparisons of the mean activity concentrations of the various radionuclides showed statistically significant differences. However, the p-value obtained for the hardened paste cube made with 20% LFS compared to the ground cube indicated that there were statistically significant differences. These results allowed for the formulation of various conclusions. Firstly, the activity concentrations of radionuclides in the hardened cubes and ground cubes were equivalent, confirming the homogeneity of the mixtures and corroborating that both the measurement of the cubes and the polypropylene boxes containing the powdered solids yielded the same results. Secondly, the results for LFS were not comparable, which could indicate possible heterogeneity in this type of SCM, leading to the ^234^Th activity concentration having an RSD (%) of 23.21%. This significant difference could be attributed to the influence of self-absorption, as at energies below 200 keV, the photoelectric effect is highly pronounced (Kajal et al. 2023), and this effect is strongly dependent on the homogeneity of the sample. Thirdly, no differences were observed between carbonated and non-carbonated SCMs in either the hardened paste cubes or the powdered samples obtained from them. This finding, along with the fact that the pastes (both in cube form and ground cubes) were statistically comparable to OPC, allows the conclusion that, from a radiological perspective, the cubes are not affected by the addition of the three carbonated alkaline SCMs. Therefore, despite the significant difference between ^40^K and ^210^Pb in the pastes made with BA and BAc, these differences are not sufficient to conclude that the activity concentrations of the analysed radionuclides were statistically different.

### Annual effective dose rates

**Fig. 3:**
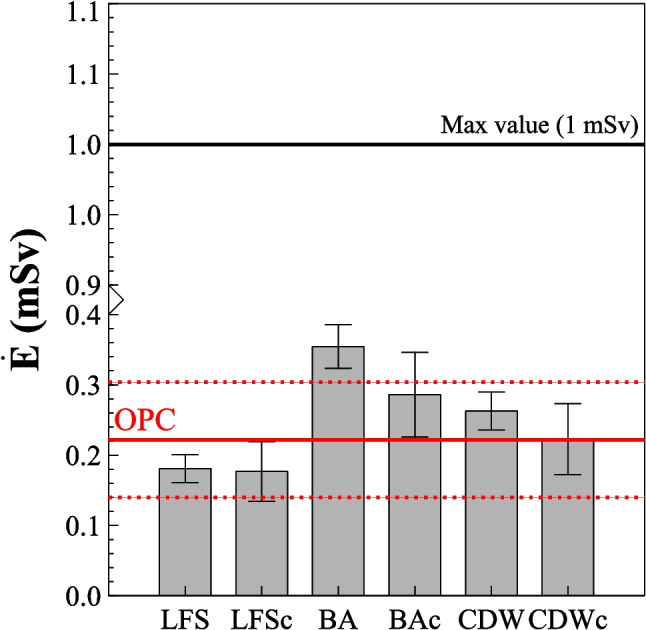
Activity concentration index (ACI) obtained for the hardened cement pastes prepared with 80% OPC and 20% supplementary cementitious material (LFS, BA, and CDW), both carbonated and non-carbonated. The ACIs were compared with the ACI of OPC, considered as the reference sample

The annual effective dose rate ($\dot{{E}_{g}}$, mSv) was determined for the final construction material as established in Annex VIII of the European Directive 2013/59 (EU 2013). For this purpose, the absorbed dose rates ($\dot{D}$) were calculated using the activity concentrations obtained through gamma spectrometry and presented in Table 2. The absorbed dose rates were all below the 84 nGy h^−1^ of natural background radiation (UNSCEAR 2008). Figure 3 shows the effective dose rates from external radiation for the hardened paste cubes prepared using 80% OPC and 20% supplementary cementitious material (SCM): LFS, BA, and CDW, both carbonated and non-carbonated. The results obtained indicate that the ${E}_{g}$ values are statistically equivalent to those determined for OPC, except for the hardened paste cube prepared with 80% OPC and 20% BA. This outcome is due to the ^40^K activity concentration in BA being 67% higher than that in OPC. However, the value for BAc overlaps with that of BA, suggesting that the observed difference compared to OPC is more likely related to measurement uncertainty rather than a potential loss of ^40^K during the accelerated carbonation process. Additionally, the results showed that all *E*_g_ values were well below the 1 mSv per year threshold, and all were below the effective dose rate of 0.48 mSv, which corresponds to natural background radiation. Therefore, cement pastes manufactured with 80% OPC and 20% SCM are safe from the perspective of radiological protection concerning external radiation.

### Radon emanation and exhalation

**Fig. 4:**
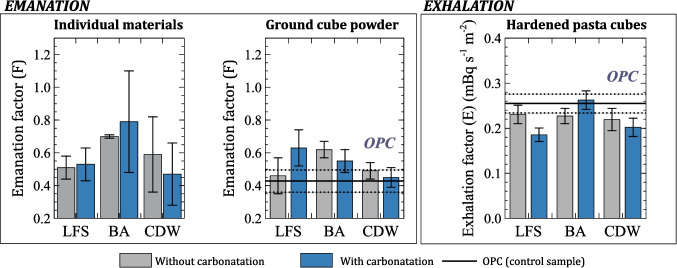
Emanation factors (*F*) of individual materials and ground cubes, along with the exhalation factor (*E*, Bq s^−1^ m^−2^) of hardened paste cubes fabricated with 80% OPC and 20% SCMs (LFS, BA, and CDW). The solid line represents the value obtained for paste cubes made with 100% OPC, and the dashed lines represent the uncertainties associated with *F* and *E* for OPC

Figure 4 presents the emanation factors (*F*) of powdered solids from individual materials and those from ground hardened paste cubes. Additionally, the figure includes the exhalation factors (*E*, mBq s^−1^ m^−2^) of 5 × 5 × 5 cm^3^ cubes prepared with 80% OPC and 20% SCMs (LFS, BA, and CDW), both with and without undergoing the carbonation process. The graphs for the ground cubes and hardened cubes were compared with the emanation and exhalation factor values obtained for the OPC (100%) sample, considered as the control sample. The comparison with the OPC was not made regarding the alkaline samples (individual materials) as carbonation did not occur in the anhydrous cement.

There is a lack of literature data on emanation factors for the three types of SCMs used in this study. However, the *F* values obtained for CDW are equivalent to those reported for cements and aggregates by Keller et al. (Keller et al. 2001), who found emanation factor values of 0.15 for OPC cement and 0.30 for the aggregates used. The results obtained for the ^222^Rn emanation factor reveal two important aspects. Firstly, carbonation does not significantly affect the *F* values obtained, as all results overlapped for both carbonated and non-carbonated samples, within a coverage factor of *k* = 2. Secondly, the comparison of the *F* values for the powder obtained after grinding hardened paste cubes fabricated with 20% SCMs showed equivalence to the *F* value obtained for OPC, except for LFSc and BA. However, these differences can be attributed to measurement uncertainty, as the ^226^Ra activity concentrations are low for these types of materials, as observed in Table 2. Finally, the *F* values obtained for both the individual materials and the ground cubes overlapped within a coverage factor of *k* = 2, as shown in Fig. 4.

The exhalation factors (*E*, mBq s^−1^ m^−2^) obtained for hardened paste cubes fabricated with 20% LFS, BA, and CDW, both with and without accelerated carbonation, were comparable to those obtained for OPC. In this regard, the *E* value obtained for the OPC cube fabricated with CEM I 52.5 R cement was consistent with the 0.83 ± 0.13 Bq m^−2^ h^−1^ reported for a cube of the same cement at 28 days in a previous study (Castaño-Casco et al. 2025). Similarly, these results align with those obtained by Kovler et al. (Kovler 2007) for CEM I 52.5 R cements. On the other hand, Yang et al. (Yang 2022) examined the influence of radon exhalation during the ageing of construction materials through accelerated carbonation, concluding that this process could increase ^222^Rn exhalation by a factor of 1.5 compared to values obtained after 2 days (Yang et al. 2023). Although that study was conducted on final materials without the addition of alkaline waste SCMs, the final exhalation values were similar to those obtained in this study. Additionally, the *E* results shown in Fig. 4 were also comparable to those obtained for concrete samples, with values of 0.46 ± 0.15 mBq m^−2^ s^−1^ (0.255 ± 0.021 mBq m^−2^ s^−1^ in this study) (Keller et al. 2001; Steiner et al. 2005). Regarding pastes made with these types of materials, only studies on pastes fabricated via alkali activation with steel slag are available (Sas et al. 2019), which reported values of the same order of magnitude but lower. However, alkali-activated pastes differ in their microstructure due to the formation of gels other than the C-S-H found in OPC, such as N-A-S-H, C-A-S-H, or C-N-A-S-H. For this reason, these results are not directly comparable and do not allow conclusions to be drawn about whether there is an increase or decrease in ^222^Rn exhalation. Therefore, the results obtained demonstrate that the use of these alkaline waste materials does not increase the exhalation of ^222^Rn and does not alter the microstructure compared to other types of pastes. This finding confirms the microstructural changes observed in pastes fabricated with LFSc, BAc, and CDWc in previous studies (Moreno de los Reyes et al. 2025). The results of this study showed a change in the shape and number of pores, with a reduction in larger pores and an increase in finer pores. This structural change results in a more compact microstructure, equivalent to that of pastes made with 100% OPC. Figure 4 shows that the exhalation values obtained for pastes with LFSc and CDWc are lower than those for the other materials. This effect could be attributed to the rearrangement of pores within the microstructure. However, a Student’s *t*-test comparing the exhalation of the cube made with 100% OPC to those made with 20% SCMs yielded a *p*-value of 0.25, indicating no statistically significant differences. Therefore, it would be necessary to test a broader set of these additions to obtain more reproducible conclusions.

## Conclusions

The results obtained in this study have confirmed that carbonated alkaline materials, despite containing high activity concentrations of natural radionuclides such as ^40^K and ^210^Pb, do not exceed an annual effective dose rate from external radiation higher than 1 mSv, and both the emanation and exhalation of ^222^Rn are equivalent to those of OPC cement. The activity concentrations of natural radionuclides were below 40 Bq kg^−1^ except for BA and BAc, which showed activity concentrations for ^40^K and ^210^Pb between 4000 and 5000 Bq kg^−1^. Additionally, this type of material also exhibited ^137^Cs activities of 30 Bq kg^−1^, which, although unusual in construction materials, do not pose a health risk. The higher presence of ^40^K and ^210^Pb is due to the radionuclide concentration process that occurs during the generation of ash from the burning of vegetation. BA and BAc were the only individual materials that showed statistically significant differences, although no chemical justification for this difference was found. However, the observed imbalance between ^226^Ra and ^214^Pb was likely caused by a chemical process, as previous studies have shown a decrease in CaO during the carbonation process of LFSc and BAc. Despite these activity concentrations, the annual effective dose rates remained below 1 mSv, with absorbed dose rates below the natural background radiation level of 84 nGy h^−1^. Therefore, it has been confirmed that the external radiation dose from these materials complies with European Directive 2013/59.

The next aspect considered to verify the radiological suitability of these materials was the determination of ^222^Rn emanation and exhalation. The values obtained for ^222^Rn emanation had a mean value of 0.6, which was equivalent to those obtained for the ground hardened paste cubes. Similarly, the emanation values of the ground cubes fabricated with SCMs derived from alkaline waste were equivalent to those of the cube prepared with 100% OPC cement, which was used as a control sample. The emanation values obtained for the ground cubes with carbonated and non-carbonated alkaline waste SCMs were statistically identical for a coverage factor of *k* = 2. Finally, the exhalation values obtained for the hardened paste cubes prepared with 20% LFS, BA, and CDW (both with and without accelerated carbonation) were equivalent to those of OPC cement and even slightly lower. This could be explained by the pore rearrangement that occurs in hardened cement pastes with the addition of carbonated alkaline wastes. Therefore, the materials tested in this study are safe from the perspective of radiological protection.

## Data Availability

Raw data that support the findings of this paper are available from the corresponding author upon request.

## Declarations

### Clinical trial number

This is not applicable.
